# fMRI investigation of response inhibition, emotion, impulsivity, and clinical high-risk behavior in adolescents

**DOI:** 10.3389/fnsys.2015.00124

**Published:** 2015-09-29

**Authors:** Matthew R. G. Brown, James R. A. Benoit, Michal Juhás, Ericson Dametto, Tiffanie T. Tse, Marnie MacKay, Bhaskar Sen, Alan M. Carroll, Oleksandr Hodlevskyy, Peter H. Silverstone, Florin Dolcos, Serdar M. Dursun, Andrew J. Greenshaw

**Affiliations:** ^1^Department of Psychiatry, University of AlbertaEdmonton, AB, Canada; ^2^Department of Computing Science, University of AlbertaEdmonton, AB, Canada; ^3^Psychology Department, Neuroscience Program, and the Beckman Institute for Advanced Science and Technology, University of Illinois Urbana-ChampaignUrbana, IL, USA

**Keywords:** response inhibition, high-risk behavior, impulsivity, emotional Go/NoGo, adolescent, ARBS

## Abstract

High-risk behavior in adolescents is associated with injury, mental health problems, and poor outcomes in later life. Improved understanding of the neurobiology of high-risk behavior and impulsivity shows promise for informing clinical treatment and prevention as well as policy to better address high-risk behavior. We recruited 21 adolescents (age 14–17) with a wide range of high-risk behavior tendencies, including medically high-risk participants recruited from psychiatric clinics. Risk tendencies were assessed using the Adolescent Risk Behavior Screen (ARBS). ARBS risk scores correlated highly (0.78) with impulsivity scores from the Barratt Impulsivity scale (BIS). Participants underwent 4.7 Tesla functional magnetic resonance imaging (fMRI) while performing an emotional Go/NoGo task. This task presented an aversive or neutral distractor image simultaneously with each Go or NoGo stimulus. Risk behavior and impulsivity tendencies exhibited similar but not identical associations with fMRI activation patterns in prefrontal brain regions. We interpret these results as reflecting differences in response inhibition, emotional stimulus processing, and emotion regulation in relation to participant risk behavior tendencies and impulsivity levels. The results are consistent with high impulsivity playing an important role in determining high risk tendencies in this sample containing clinically high-risk adolescents.

## 1. Introduction

Adolescence is a period of increasing high-risk behavior for many individuals. Examples of high-risk behavior include alcohol binging, substance abuse, unsafe sex, physical violence, physical risk-taking, and self-harm. At a population level, high-risk behavior tendencies start to increase between ages 13–17 (Statistics Canada, 2010a,b; Viner et al., 2012). Many risk behaviors such as binge drinking and impaired driving peak between ages 20–24 and then decline (Statistics Canada, 2010a,b). Although many important risk behaviors peak later in life, engagement in these behaviors begins to increase in adolescence, creating attendant risks for poor outcomes. For example, deaths due to suicide and accidental injury peak in the 50–54 and 90+ age ranges, respectively (Statistics Canada, 2010a,b). Nonetheless, suicide and accidental injury consistently remain the two leading causes of death in persons aged 15–21 in Canada, with rates higher for males than females (Statistics Canada, 2010b). In 2004, suicide was the third leading cause of death among persons aged 10–24 years in the United States (NCHS, 2007). High-risk behaviors in adolescence and young adulthood are also associated with poor physical and mental health outcomes in later life (Anda et al., 2006; Eaton et al., 2006; Hawton and O'Connor, 2012). Moran et al. (2012) have reviewed self-harm in adolescence and point to the need to understand the underlying factors for this vulnerable age group. Such understanding is also important given that self-harm is an indicator of future mental health problems (see also Hawton and O'Connor, 2012). Apart from our shared concern in terms of human suffering, the economic implications of an increased health system burden related to neurodevelopmental challenges are staggering (Stephens and Joubert, 2001). Research that can inform us about mechanisms of brain function that underlie high-risk behavior in adolescents and young adults is very important and has implications for adolescent and subsequent mental health, health services delivery, and health policy.

The population-level risk patterns described are comprised of subgroups with different risk behavior patterns, which likely have different etiologies (Romer, 2010). First, it is important to note that many adolescents do not engage in high-risk behavior to any great degree. In a study of adolescent binge drinking, Hill et al. (2000) reported that the majority (70%) of adolescents age 13–18 did not binge drink. 23% began binge drinking around age 16 and increased the frequency of binge drinking into young adulthood. A small minority (4%) began binge drinking earlier, around age 14–15, and exhibited increasing and substantially higher binge drinking frequencies into young adulthood. Another small minority (3%) exhibited a very early onset of binge drinking before age 13 combined with a peak at age 14–15 followed by declining binge drinking into young adulthood. One of this study's conclusions is that a small minority of individuals account for a substantial proportion of adolescent binge drinking. A study of aggressive behavior in children and adolescents (Nagin and Tremblay, 1999) supports a similar conclusion with regard to physical aggression.

The interaction of emotion and executive control of behavior is a crucial focal point for understanding the neural basis of decision making in high-risk situations such as those involving drug use or self-harm. Explanations of high risk behavior tendencies have emphasized individual differences and developmental changes in emotion processes, reward processing, and executive control of behavior and emotions (Jessor, 1991; Arnett, 1992, 1994, 1996; Ernst et al., 2006; Steinberg, 2007; Casey et al., 2008; Ernst and Mueller, 2008; Gullo and Dawe, 2008; Steinberg, 2008; Ernst and Fudge, 2009; Romer et al., 2009; Romer, 2010; Casey et al., 2011; Dalley et al., 2011; Mitchell, 2011; Blakemore and Robbins, 2012; Whelan et al., 2012).

We used an emotional Go/NoGo task with functional magnetic resonance imaging (fMRI) to investigate processes related to response inhibition and emotion processing in a sample of 21 adolescents (age 14–17 years) with a range of risk behavior tendencies. Risk behavior was assessed using the Adolescent Risk Behavior Screen (ARBS; Jankowski et al., 2007). Our sample included participants recruited from pediatric psychiatry clinics with high ARBS risk scores. We used a variant of the classic Go/NoGo task (Donders, 1868/1969). Our task presented emotionally neutral or aversive distractor pictures simultaneously with Go/NoGo stimuli. Participants had to ignore the distractors, respond to Go stimuli by pressing a button, and respond to NoGo stimuli by withholding the button press. Go trials were 4 times as frequent as NoGo trials to increase prepotency of Go trials. See Section 2.3 for task details.

Our group has previously reported fMRI results using this emotional Go/NoGo task with young adult participants (Brown et al., 2012). Brown et al. (2012) found differences in fMRI activation related to response-inhibition and emotion processing in multiple regions in all lobes of the brain. Activation in left motor cortex and other regions was significantly larger for Go vs. NoGo trials. In an analysis of response inhibition (NoGo vs. Go), ventrolateral prefrontal (vlPFC) cortex as well as other cortical regions exhibited larger activation for NoGo compared to Go trials. These findings are consistent with previous Go/NoGo studies (Garavan et al., 1999, 2002; Watanabe et al., 2002; Mostofsky et al., 2003; Aron et al., 2004b; Fassbender et al., 2004; Kelly et al., 2004; Rubia et al., 2005; Wager et al., 2005; Aron et al., 2007b; Mitchell, 2011). In an analysis of emotional valence (aversive vs. neutral distractors), greater activation for aversive distractor pictures was displayed in orbitofrontal cortex, lateral prefrontal cortex, insula, the amygdala and surrounding cortex, anterior cingulate cortex, medial prefrontal cortex, and bilateral posterior middle temporal gyrus and angular gyrus. These results are also consistent with previous fMRI studies of emotional picture processing (Irwin et al., 1996; Bermpohl et al., 2006; Meseguer et al., 2007). Brown et al. (2012) investigated interactions of response inhibition and emotion processing in vlPFC by emotional context. These two sources of fMRI activation changes summated in a straight-forward manner; emotional context (aversive vs. neutral distractors) did not suppress or potentiate fMRI signals related to response inhibition in vlPFC. See Brown et al. (2012) for further details.

More broadly, the results of Brown et al. (2012) are consistent with the literature on response inhibition, cognitive control, emotion processing, and emotion regulation. Dorsolateral prefrontal cortex (dlPFC), vlPFC, orbitofrontal cortex (OFC), and ventromedial PFC (vmPFC) are involved in response inhibition in the Go/NoGo task as well as inhibition in other executive control tasks (see Aron et al., 2004a, 2007b; Chikazoe, 2010; Dolcos et al., 2011; Mitchell, 2011; Mahmood et al., 2013). Anterior cingulate cortex (ACC) has been implicated in error detection and conflict monitoring in the Go/NoGo and other cognitive tasks (Carter et al., 1998, 1999; Garavan et al., 1999; Botvinick et al., 2004; Kerns et al., 2004; Brown and Braver, 2005; Mitchell, 2011). Dorsomedial PFC (dmPFC) may also contribute to response conflict processing (see Ridderinkhof et al., 2004) as well as to response selection and response inhibition in the Go/NoGo task (Simmonds et al., 2008). dmPFC is also thought to be involved in resolution of response conflict and outcome value-related aspects of decision making (Venkatraman et al., 2009). There are suggestions that primary motor and premotor cortex may be involved in response inhibition, in addition to generation of motor responses (see Coxon et al., 2006; Mirabella et al., 2011; Mattia et al., 2012, 2013). OFC and vlPFC are thought to be involved in processing emotional stimuli, for example to evaluate valence (Dolcos et al., 2011; Mitchell, 2011). Multiple prefrontal regions including OFC, vmPFC, dmPFC, vlPFC, and dlPFC are also associated with emotion regulation (Dolcos et al., 2011; Mitchell, 2011; Golkar et al., 2012).

We are aware of one other fMRI study using an emotional Go/NoGo task with adolescent participants (Hare et al., 2008). In this study, the emotional valence of the stimulus served as the Go/NoGo signal, necessitating a blocked aspect in the trial design to accommodate different stimulus valence to Go/NoGo assignments. For example, Hare et al. (2008) presented specific valence to Go/NoGo assignments in different functional runs. This approach introduces potential extraneous task set effects—neutral Go and aversive NoGo trials occur in the context of one assignment; aversive Go and neutral NoGo trials occur in the context of a different assignment. In contrast, we presented emotional distractors with non-emotional Go/NoGo stimuli, such that task performance did not require use of any information from the distractors. This design choice allowed us to interleave all trial types in the same task set context, resulting in cleaner methodological and logical separation of differences related to NoGo vs. Go effects and aversive vs. neutral distractor effects.

We characterized participant impulsivity using the Barratt Impulsivity Scale (BIS; Barratt, 1959) and compared fMRI results against both ARBS risk scores and Barratt impulsivity scores. Individual differences in impulsivity and impulse control are thought to play a role in high-risk behavior. Higher impulsivity scores on psychometric questionnaires have been associated with increased risk behavior tendencies (Levitt, 1991; Moore and Rosenthal, 1993; Luengo et al., 1994; Stanford et al., 1996; Gullo and Dawe, 2008; Romer et al., 2009; Romer, 2010; Dalley et al., 2011; Mishra and Lalumière, 2011; Christiansen et al., 2012). The relationship between impulsivity and high-risk behavior is complex (Romer, 2010; Dalley et al., 2011; Blakemore and Robbins, 2012), and some aspects of high-risk behavior do not seem to be associated particularly strongly with impulsivity (Steinberg et al., 2008; Romer, 2010; Brown et al., 2015). However, certain aspects of dangerous high-risk behavior do seem to be associated with elevated impulsivity (see Romer, 2010). The inclusion of BIS scores allowed us to investigate potential relationships between high-risk tendencies and impulsivity.

One study reported increased activation on NoGo trials in left dmPFC, right ACC, right dlPFC, and left precentral gyrus in adolescent participants with internet gaming addiction compared to controls (Ding et al., 2014). Impulsivity scores from the BIS were also associated with internet gaming addiction and left dmPFC activation in that study. Goldenberg et al. (2013) found that risky sexual tendencies in adolescent participants were inversely related to response inhibition fMRI activation in a Go/NoGo task evoked in multiple brain regions in left and right dlPFC, vlPFC, and insula. In adults, impulsivity measures have been associated with differences in fMRI activation patterns in the Go/NoGo task. Greater BIS scores were associated with less response inhibition-related fMRI activation in right dlPFC (Asahi et al., 2004) and in dmPFC (Horn et al., 2003). Horn et al. (2003) also reported a positive correlation between Eysenck's Impulsivity Scale and response inhibition-related activation in right vlPFC. The literature suggests, then, that many prefrontal regions may be involved in individual differences in impulsivity levels and risk tendencies, although different papers implicate somewhat different sets of specific regions. The precise relationships between brain functions related to behavioral control, emotion representation, and emotion regulation in various prefrontal regions, fMRI activation patterns, and individual impulsivity and high-risk behavior tendencies constitute an open area of research.

### 1.1. Hypotheses

Given the discussion above and the well-known involvement of prefrontal cortex (PFC) in executive control, emotion processing, and emotion regulation (Rubia et al., 2001; Aron et al., 2004a, 2007b; Chikazoe, 2010; Dolcos et al., 2011; Mitchell, 2011; Mahmood et al., 2013), we expected that one or more prefrontal regions would show a relationship between participant ARBS risk scores and/or BIS impulsivity scores and task-related fMRI activation patterns, as specified in the following hypotheses:

- **Hypothesis 1a:** One or more prefrontal regions will exhibit an association between activation levels related to response inhibition and participant risk and/or impulsivity scores.
- **Hypothesis 1b:** One or more prefrontal regions will exhibit an association between activation levels related to emotion processing and participant risk and/or impulsivity scores.
- **Hypothesis 1c:** One or more prefrontal regions will exhibit an association between activation patterns reflecting the interaction of response inhibition and emotion processing^1^ and participant risk and/or impulsivity scores.

Due to the large number of different prefrontal regions previously reported to show differences in response inhibition based on participant impulsivity or risk tendencies (see discussion above and Horn et al., 2003; Asahi et al., 2004; Goldenberg et al., 2013; Ding et al., 2014), we did not constrain Hypotheses 1a–1c to specific prefrontal brain regions. That is, these hypotheses were specific in predicting associations between task-related prefrontal fMRI changes with risk and/or impulsivity scores but exploratory in terms of which prefrontal brain regions would exhibit modulation by risk and/or impulsivity scores. Despite the broad prefrontal focus, vlPFC, especially on the right side, was of particular interest, given the well-replicated finding that fMRI activation in vlPFC is associated with response inhibition (see Chikazoe, 2010). We also did exploratory analyses of potential relationships between task-related fMRI activation patterns and risk and/or impulsivity scores and brain regions outside prefrontal cortex.

We sought to address the specific question of how high impulsivity is related to high risk behavior. Despite previous associations between impulsivity and high risk behavior tendencies (Levitt, 1991; Moore and Rosenthal, 1993; Luengo et al., 1994; Stanford et al., 1996; Gullo and Dawe, 2008; Romer et al., 2009; Romer, 2010; Dalley et al., 2011; Mishra and Lalumière, 2011; Christiansen et al., 2012), recent results from our group suggest a possible dissociation between impulsivity and risk behavior (Brown et al., 2015). Therefore, we did not have an *a priori* prediction about whether risk and impulsivity scores would have similar or distinct associations with differences in prefrontal fMRI activations. We anticipated that either outcome would contribute to this open question. We formulated this question as two opposing hypotheses:

- **Hypothesis 2a:** Risk and impulsivity scores will exhibit similar associations with fMRI activation patterns; brain regions exhibiting an association between fMRI activation patterns and participant risk tendencies will show similar associations between fMRI activation patterns and participant impulsivity scores.
- **Hypothesis 2b:** There will be a dissociation between risk and impulsivity scores; different brain regions will show relationships between fMRI activation and risk scores compared to fMRI activation and impulsivity scores.

We also tested predictions related to two models of high-risk behavior in adolescents. The Triadic Model of Ernst and colleagues (Ernst et al., 2006; Ernst and Mueller, 2008; Ernst and Fudge, 2009) suggests that elevated adolescent risk tendencies (relative to younger or older age groups) are caused in part by developmental changes in emotion-related processing in limbic regions. Specifically, the Triadic Model posits increased approach signals, including signals driven by reward or other emotionally positive stimuli, in the nucleus accumbens and other limbic regions in adolescents. It also posits decreased harm-avoidance signal, including signals driven by emotionally aversive stimuli, in the amygdala and other limbic regions in adolescents. In addition, the Triadic Model attributes elevated adolescent risk behavior partially to incomplete development of prefrontal regulatory functions. Though this model does not address individual differences in risk behavior among adolescents (Nagin and Tremblay, 1999; Hill et al., 2000; Berns et al., 2009; Romer, 2010), we suggest that this model would be more consistent with reduced limbic fMRI responses to aversive distractors in high risk participants (Hypothesis 3a below) as well as reduced activation related to response inhibition in prefrontal regions in high risk participants (Hypothesis 4 below). The model of Casey et al. (2008, 2011) attributes elevated adolescent risk behavior in part to elevated emotional responses in limbic regions, including the amygdala, as well as to incompletely developed prefrontal regulatory function. We propose that this model would be more consistent with increased limbic responses to aversive distractors in high risk participants (Hypothesis 3b below, opposite to the Ernst Triadic Model's prediction) as well as reduced response inhibition activation in prefrontal regions in high risk participants (Hypothesis 4 below, identical to the Ernst Triadic Model). We investigated fMRI responses to the distractor images in limbic regions including the amygdala, as well as possible modulation based on participant risk and impulsivity levels. We did not have a prediction about which of the two models would be better supported. This question is formulated as two opposing hypotheses:

- **Hypothesis 3a:** Limbic regions including the amygdala will show reduced emotion response activation in participants with higher risk scores, consistent with the Ernst Triadic Model.
- **Hypothesis 3b:** Limbic regions including the amygdala will show increased emotion response activation in participants with higher risk scores, consistent with the Casey Model.

We also investigated differences in prefrontal response inhibition activation related to risk tendencies. Our prediction based on the discussion above was that higher risk participants would exhibit reduced activation related to response inhibition in one or more prefrontal regions, with a particular focus on vlPFC:

- **Hypothesis 4:** Prefrontal regions including vlPFC will exhibit reduced response inhibition activation in participants with higher risk scores, consistent with the Ernst Triadic Model and the Casey Model.

## 2. Methods

The Health Research Ethics Board at the University of Alberta approved this study.

Due to substantial similarities between the methods described here and those of Brown et al. (2015), from our research group, parts of the methods descriptions below were adapted from that paper.

### 2.1. Participants

Twenty-one adolescents were recruited into the study (13 female and 8 male, age range 14.8–17.7 years, mean age 16.0 ± 1.1 years). Based on the Edinburgh Handedness Inventory (Oldfield, 1971), 19 participants were right-handed, and two participants were ambidextrous. All participants gave informed, written assent in English, and a parent or guardian gave informed, written consent. Participants did not receive any financial incentive to participate in the study. They were given a Tim Horton's gift card worth $10 at the end of the study as a thank-you. The gift card did not provide any incentive toward study participation or task performance as participants were not told they would receive it at any time before receiving it.

Fourteen participants were recruited from the general population. These participants reported no history of psychiatric disorder, neurological disorder, or learning disability, and they were not taking any psychoactive medication. Three of these fourteen participants had ARBS risk scores in the high-risk range (≥17, see Questionnaires Section 2.2 below). The other 11 participants had ARBS scores in the low-risk range (≤13, see Questionnaires Section 2.2 below).

To enable investigation of clinical high-risk behavior, we also recruited seven participants with high-risk behavior tendencies from pediatric psychiatry clinics in the Edmonton area. These participants did not report any history of neurological disorder. One reported no history of psychiatric diagnosis and was not on psychoactive medication. The other six participants reported some history of psychiatric symptoms (see Table 1). All seven of these participants had ARBS scores ≥17.

**Table 1:** **Psychiatric symptoms**.

**No**.	**Psychiatric symptoms**	**Psychoactive medication**
1	Depression, personality disorder, ADHD,	Melatonin to help with sleep
	possible learning disability	
2	Possible learning disability	None
3	Mood disorder	None
4	Anxiety, depression	Fluoxetine, pericyazine as needed
5	Depression, ADHD	Bupropion, risperidone, pericyazine
6	ADHD, possible learning disorder	Atomoxetine
7	None	None

*Psychiatric symptoms reported by the seven participants recruited from pediatric psychiatric clinics*.

### 2.2. Questionnaires

Participant risk behavior tendencies were characterized with the Adolescent Risk Behavior Screen (ARBS; Jankowski et al., 2007). This provides a score from 9 (lowest risk) to 30 (highest risk). Jankowski et al. (2007) suggest a cutoff of >17 for defining high-risk status. We used an ARBS cutoff of ≥17 as our participants exhibited a natural break with ARBS scores being either ≤ 13 or ≥17.

To assess participants' impulsivity, we used the Barratt Impulsivity Scale, version 11 (BIS; Patton et al., 1995). The BIS includes six first order subscales: attentional, cognitive instability, motor, perseverance, self-control, and cognitive complexity. We took the sum over all 30 questions in the BIS (after reversing scores for appropriate items) as a participant's impulsivity score. This is equivalent to taking the sum of the first order subscale scores. Overall BIS scores can range from 30 (least impulsive) to 120 (most impulsive). BIS scores from 52 to 71 represent a normal range of impulsivity, with scores at or below 51 indicating a very controlled, non-impulsive individual and scores at or above 72 representing a highly-impulsive individual (Stanford et al., 2009).

### 2.3. Task

**Figure 1:**
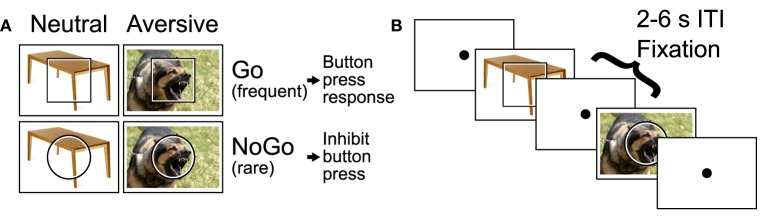
**Emotional Go/NoGo task**. **(A)** Each trial was either a Go or NoGo trial and featured an emotionally neutral or aversive distractor picture. **(B)** Example segment of two trials with 2–6 s fixation intertrial intervals (ITIs) interleaved. Figure reproduced from Brown et al. (2012).

We employed an emotional Go/NoGo task (see Donders, 1868/1969; Hester and Garavan, 2004), which presented emotional distractor pictures simultaneously with the Go and NoGo stimuli. In each trial, the participant was shown a square or circle, lasting 2 s, which served as the Go or NoGo stimulus (see Figure 1). The assignment of shape to trial type was counterbalanced across participants. Each Go or NoGo stimulus was superimposed on a task-irrelevant distractor image. Each distractor image was either emotionally neutral or aversive. Distractor images were taken from the International Affective Pictures System (IAPS) (Lang et al., 2008). On Go trials, the participant had to press a button with their right index finger. On NoGo trials, the participant had to withold the button press response. To make the Go response more automatic (prepotent), Go and NoGo trials were presented at a 4:1 ratio. The task included four trial types: neutral Go, neutral NoGo, aversive Go, and aversive NoGo. Between trials, participants fixated a dot located at screen centre (Figure 1B). Participants were asked to perform each trial quickly and accurately.

IAPS images were chosen as follows. IAPS images were screened by two child psychiatrists to be acceptable for use with adolescent psychiatric participants. From the screened images, aversive and neutral distractor pictures were selected based on the IAPS measures of valence and arousal from the normative sample reported in Lang et al. (2008). To maximize the effect of distractor valence, we used image selection criteria that created two non-overlapping clusters of images in two-dimensional arousal-valence space, one cluster for aversive distractors and one for neutral distractors (see Supplementary Figure 1). Specifically, we selected the 100 aversive IAPS images that had valence ratings ≤ 3.6 and were closest to [arousal, valence] target position [9, 1]. Position [9, 1] represents the most aversive (lowest valence rating), most arousing possible score. We selected the 104 neutral images with valence ratings >3.6 and < 6.4 that were closest to [arousal, valence] target position [1, 5], which represents a neutral valence and the smallest possible arousal score. It would have been preferable to match distractor images for scene complexity, number of objects, and so on across the different trial types. Unfortunately, the IAPS set did not include enough images to permit such matching while also satisfying the above-described criteria: screening by psychiatrists and separation into two non-overlapping clusters (as can be seen in Supplementary Figure 1). Aversive distractor pictures presented a variety of scenes including threatening animals, aggressive human faces, individuals wielding guns in a threatening manner, human injuries, surgical scenes, vehicle accidents, terrorism-related scenes, individuals vomiting, and dirty toilets including feces.

Trials were presented in a rapid event-related design. Each Go or NoGo trial lasted one volume, i.e. 2 s. Inter-trial intervals were pseudo-randomized from the set {2, 4, 6 s}, distributed 30% 2 s, 40% 4 s, 30% 6 s with a mean of 4 s. Trial sequences and timings were derived using custom Python code to ensure linear independence of trial activations (see Burock et al., 1998). First-order counterbalancing of trial sequences was used to avoid first-order interaction effects between adjacent trials. To avoid interaction of BOLD non-linearity with inter-trial intervals and trial types, each of the four trial types was preceded in equal proportions by the 2, 4, and 6 s inter-trial intervals. Participants each completed four 330 s functional runs with a combined total of 204 trials including 84 neutral Go trials, 80 aversive Go trials, 20 neutral NoGo trials, and 20 aversive NoGo trials. The first trial of every run was always a neutral Go trial.

### 2.4. MRI scanning

Magnetic resonance imaging was done on the 4.7 Tesla Varian Inova scanner at the Peter S. Allen MR Research Centre at the University of Alberta. We acquired blood oxygenation level dependent (BOLD) fMRI images with a T2^*^-weighted echo planar imaging sequence using these parameters: volume time 2.0 s, single shot, repeat time 2.0 s, echo time 19.0 ms, 3.0 mm isotropic voxels, 80 × 80 matrix, 240 × 240 mm^2^ field of view, 3.0 mm slice thickness, 36 axial slices, 108 mm through-plane coverage, interleaved slice collection order. We used 80% partial k-space in the phase encode direction (anterior-posterior). The fMRI scanning volume covered the entire cerebral cortex except for the ventral-posterior tip of occipital cortex in participants with larger heads. A high resolution T1-weighted structural scan was also acquired for each participant. This scan utilized a magnetization-prepared rapid acquisition gradient echo (MPRAGE) sequence with parameters: TR 9.4 ms, inversion time 300.0 ms, relaxation delay time (after readout prior to inversion) 300.0 ms, linear phase encoding, TE 3.7 ms, matrix 240 × 192 × 128, field of view 240 × 192 × 192 mm^3^, 1.0 × 1.0 × 1.5 mm^3^ voxels, whole brain coverage.

### 2.5. Analysis of task performance and questionnaires

Error rates were not normally distributed (heavily skewed toward zero). Bootstrap and permutation tests were used to compare NoGo commission error rates vs. zero and commission rates for aversive vs. neutral NoGo trials. *T*-tests were used to compare Go trial latencies vs. zero and to compare neutral vs. aversive Go trial latencies. A participant's overall Barratt impulsivity score was computed as the sum of the six BIS subscales: attentional, cognitive instability, motor, perseverance, self-control, and cognitive complexity. We did a correlation analysis of ARBS risk vs. BIS impulsivity scores. Bootstrap regression tests (resampling residuals) were used to compare NoGo error rates against ARBS and BIS scores. Standard linear regression was used to compare Go trial latencies against ARBS and BIS scores.

### 2.6. fMRI analysis

SPM8 and in-house MATLAB code were used for preprocessing of fMRI data. The preprocessing steps for each participant included: (1) 6 parameter rigid body motion correction of fMRI volumes, (2) non-linear spatial warping of fMRI data to the MNI EPI template space (interpolated to 3 × 3 × 3 mm^3^ spatial resolution), (3) 8 mm full width at half maximum (FWHM) Gaussian spatial smoothing of fMRI data, (4) non-linear spatial warping of MPRAGE structural scans to the MNI T1 template space (interpolated to 1 × 1 × 1 mm^3^ resolution). The first 6 volumes were discarded from each functional run to account for spin saturation effects.

Statistical modeling of fMRI data was done in two steps using custom-built MATLAB code. We first performed separate first-level, within-subjects general linear model (GLM) analyses on each participant. Within-subjects results were then combined using three different between-subjects mixed-effects analyses (Worsley et al., 2002). A manually-constructed mask was used to exclude voxels outside the brain. The mask included 82,244 voxels (size 3 × 3 × 3 mm) inside the brain. Each within-subject GLM included either four or five sets of finite impulse response (FIR) predictors, one set for each of the four trial types (neutral Go, aversive Go, neutral NoGo, aversive NoGo) and, for participants who made errors, one set of predictors for error trials (collapsed across trial types). Error trials were rare (0–17% of trials, per participant). The GLM included 7 FIR impulse predictors (corresponding to 7 functional volume times, each lasting 2 s) per trial type. The FIR predictors represented deconvolved activation timecourses for the different trial types (see Serences, 2004). The GLM also incorporated a set of nuisance predictors for each run. These included constant run offset, linear drift, cosine, and sine with period equal to twice the run length, and 6 rigid body motion parameters. In addition, for each run, we computed three nuisance predictors by taking the mean activation timecourse over voxels in three regions expected *a priori* to contain only noise signals: the region outside the brain, a region entirely within the white matter, and a region inside the ventricles. The region outside the brain was defined based on the above-mentioned mask. The regions inside the white matter and ventricles were defined manually using the Marsbar toolbox. We also included two nuisance predictors for each run that were derived from independent component analyses (ICA) as follows. Two ICA analyses were applied to all four fMRI runs from all 21 participants using the GIFT software package version 2.0 from Vincent Calhoun's group (http://mialab.mrn.org/software). In one analysis, the four runs for each participant were treated as separate sessions. For the other analysis, the four runs were concatenated and treated as a single session. For each analysis, 20 components were computed using the default settings. Components were visually inspected and categorized as containing task-related brain signal or noise. One component containing motion-related noise was selected from each ICA analysis, and the corresponding timecourses for each fMRI run were included as nuisance predictors in each participant's first-level GLM. Each within-subject GLM was fit to the data using weighted least squares that corrected for autocorrelated noise. Ten autocorrelation coefficients (lags of 1–10 volume times) were computed for each functional slice across the whole brain using the residuals from a non-corrected initial GLM fit. Then, the design matrix and each voxel's timecourse were pre-whitened, and auto-correlation-corrected beta weights were computed as described in Burock and Dale (2000) and Worsley et al. (2002).

For each subject separately, we computed three first-level (within-subjects) statistical contrast maps (two-tailed t statistic maps) from the GLM beta weights. The response inhibition contrast was (aversive NoGo + neutral NoGo) - (aversive Go + neutral Go). The emotional valence contrast was (aversive NoGo + aversive Go) - (neutral NoGo + neutral Go). The interaction contrast was (aversive NoGo - aversive Go) - (neutral NoGo - neutral Go). Contrasts were computed from the FIR beta weights representing activation across the 3rd and 4th time points of the FIR deconvolved timecourses. The 3rd and 4th time points, which correspond to 4 and 6 s from trial start, were chosen *a priori* based on the typical BOLD hemodynamic peak time around 4–6 s (Aguirre et al., 1998).

For each of the three first-level statistical contrasts, we performed three second-level linear regression analyses using the mixed-effects method of Worsley et al. (2002). The first second-level analysis examined mean contrast magnitude across all 21 participants for each of the three first-level contrasts. The second-level design matrix in this case consisted only of a column of ones. The other two second-level analyses modeled the linear relationship between either ARBS risk scores or BIS impulsivity scores and the first-level contrast magnitude. For these models, the second-level design matrix included one constant offset predictor column and one column with either the mean-centred ARBS scores or the mean-centred BIS scores for all 21 participants. To summarize, for the three first-level contrasts (response inhibition contrast, emotional valence contrast, interaction contrast), statistical t-maps were computed testing for significant contrast (independent of ARBS or BIS scores), significant linear relationship between contrast and ARBS scores, and significant linear relationship between contrast and BIS scores.

Statistical t-maps were thresholded voxelwise at *p* < 0.01 (|*t*| > 2.626, two-tailed, *df* = 98). A cluster size threshold of 349 voxels (9423 mm^3^) was then applied to achieve global correction for multiple comparisons at *p* < 0.05 across the voxel population. The cluster size threshold value was determined using permutation testing (Winkler et al., 2014). Briefly, we simulated a simpler version of the analysis 5000 times. On each iteration, the four trial types were randomly permuted independently for each participant. Second-level analyses were then performed using a basic GLM model (as opposed to the computationally-expensive method of Worsley et al. (2002), which would have made the simulation run time infeasible). After voxelwise thresholding at *p* < 0.01 (|*t*| > 2.863, two-tailed, *df* = 19), maximum cluster sizes were counted across the 5000 simulations. A maximum cluster size of 349 voxels or larger was found to occur in 5% of simulated analyses, and this was used as the cluster size threshold to achieve global *p* < 0.05. Note that this threshold is much larger than that computed by Monte Carlo simulation based on the method of AlphaSim (Ward, 2000). Our implementation of the AlphaSim method (fMRIMonteCluster, available at github.com/mbrown/fmrimontecluster) provides a cluster size threshold of 106 voxels for our data. This estimate was computed using a residual smoothness FWHM of 12.5 mm, which was the mean residual smoothness derived from all participants' residuals volumes using AFNI's 3dFWHMx function. Woo et al. (2014) demonstrated with fMRI analysis simulations that, with a voxelwise threshold of *p* < 0.01, the cluster size threshold returned by Gaussian random field theory can be too small to fully correct for multiple comparisons. We built a simulation similar to that presented in Woo et al. (2014) and confirmed that the cluster threshold of 349 does fully correct for multiple comparisons at the expected family-wise error rate of α = 0.05 when using a voxelwise threshold of *p* < 0.01.

In total, there were nine second-level statistical maps:

- **Map 1:** response inhibition contrast independent of ARBS or BIS,
- **Map 2:** response inhibition contrast vs. ARBS scores,
- **Map 3:** response inhibition contrast vs. BIS scores,
- **Map 4:** emotional valence contrast independent of ARBS or BIS,
- **Map 5:** emotional valence contrast vs. ARBS scores,
- **Map 6:** emotional valence contrast vs. BIS scores,
- **Map 7:** interaction contrast independent of ARBS or BIS,
- **Map 8:** interaction contrast vs. ARBS scores,
- **Map 9:** interaction contrast vs. BIS scores.

We did additional quality assurance analyses on the second-level maps described above. For each of the second-level maps, an automated algorithm was used to grow a cluster (or region) of voxels around each positive or negative statistical peak (local extremum) in the associated t-map. For a given cluster, each participant's mean BOLD signal was computed by averaging across all voxels in the cluster. First-level GLM analyses were then conducted on the average timecourses, and event-related activation timecourses were computed for each of the four trial types based on the finite impulse response predictors from the fitted GLMs. Unpublished results from our group indicate that fMRI statistical contrast maps that look acceptable on visual inspection can be generated from underlying timecourses that seem to contain substantial signal noise and that may not reflect BOLD signals from the brain. Though consideration of event-related timecourses for quality assurance purposes is not standard practice in fMRI analysis, we suggest that it should be. Accordingly, we discarded regions whose activation timecourses were severely dissimilar to the expected difference of gammas hemodynamic response function shape (see Huettel et al., 2008, ch. 7), as determined by visual inspection. Note that the regression analyses done upon the mean timecourses extracted from each region were used only for quality assurance purposes. Summary data from significant clusters (median *p*- and *t*-values for regression vs. ARBS and regression vs. BIS presented in tables in the Results section) were computed from the whole-brain statistical maps. Specifically, median *p*- and *t*-values were computed (across the voxels comprising a given cluster) from the various second-level statistical maps.

### 2.7. Correlation pattern analysis

To address Hypotheses 2a and 2b (see Section 1.1), we compared correlation relationships between ARBS risk scores and fMRI contrast values with correlation relationships between BIS impulsivity scores and fMRI contrast values. We chose 26 prefrontal regions from the Harvard-Oxford atlas (distributed with FSL, see http://fsl.fmrib.ox.ac.uk/fsl/fslwiki/Atlases). Specifically, we used the HarvardOxford-cortl-maxprob-thr25-1mm.nii file from the series of files defining the Harvard-Oxford atlas. The 26 regions are listed in the Results in Section 3.6. For each of the three first-level statistical contrasts (response inhibition, emotional valence, interaction), we computed the average contrast value for each participant for each of the 26 regions. For each of the three contrasts, for each of the 26 regions, we computed the correlation between participants' contrast values and their ARBS scores as well as between contrast values and BIS scores. This created a set of 78 correlation values for ARBS scores and another set of 78 correlation values for BIS scores. We will call the 78 correlation values from a given instrument (ARBS or BIS) that instrument's correlation pattern. The correlation pattern characterized the relationship of the instrument's scores with fMRI contrast patterns in prefrontal cortex. We present the correlation patterns computed from all 21 participants in Section 3.6.

To test whether correlation patterns for ARBS and BIS scores were similar or different, we used bootstrap sampling with 10,000 iterations. For each iteration, we randomly assigned participants into two groups with each group containing similar proportions of low-risk (ARBS score ≤ 13) and high-risk (ARBS ≥17) participants. We computed the correlation pattern for ARBS (denoted ${\vec{c}}_{a}$) using the first group and the correlation pattern for BIS (denoted ${\vec{c}}_{b}$) using the second group. We then computed the following similarity measure between the two correlation patterns $S\left({\vec{c}}_{a},{\vec{c}}_{b}\right)$:

(1) $$\begin{array}{l} S\left({\vec{c}}_{a},{\vec{c}}_{b}\right)=1-\frac{\left||{\vec{c}}_{a}-{\vec{c}}_{b}|\right|}{\sqrt{78}}. \end{array}$$

||*x*−*y*|| denotes the Euclidean distance between two vectors *x* and *y*. The $\sqrt{78}$ denominator normalizes the distance between the two length 78 vectors containing the two correlation patterns. This similarity measure takes a value of 1 when all correlations are identical and decreases as the two correlation patterns become dissimilar. A similarity measure of 0 would occur, for example, if all correlations from one pattern were 1 and all correlations from the other pattern were 0^2^.

Hypotheses 2a and 2b were tested as follows. The mean similarity measure was computed across the 10,000 iterations. A similarity measure of 0.3 or below was defined as indicating a large difference between the correlation patterns (i.e. “substantially different”). A similarity measures of 0.7 or above was defined as indicating that the correlation patterns are identical or close to identical (i.e. “very similar”). To test Hypothesis 2a, which held that risk and impulsivity measures have similar associations with fMRI patterns, we tested against a null hypothesis that the similarity measures were substantially different by computing the proportion of iterations in which the similarity measure was < 0.3. To test Hypothesis 2b, which stated that risk and impulsivity measures have different associations with fMRI patterns, we tested against a null hypothesis that the similarity measures were very similar by computing the proportion of similarity measures >0.7.

## 3. Results

### 3.1. Risk and impulsivity scores

**Figure 2:**
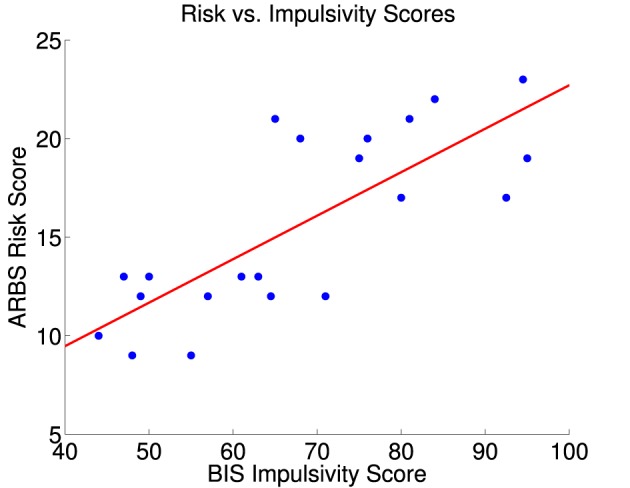
**ARBS risk scores vs. BIS impulsivity scores for 21 participants**. The red line is the best fit linear regression of ARBS scores against BIS scores. Correlation between BIS and ARBS scores was 0.78 (significant, *p* = 3 × 10^−5^, *t* = 5.42, *df* = 19). BIS scores explained 59.7% of the variance in the ARBS scores.

Mean (± standard deviation) participant ARBS risk score was 15.6 ± 4.6, and ARBS scores ranged from 9 to 23. ARBS scores range from 9 (lowest risk) to 30 (highest risk), and Jankowski et al. (2007) recommend a cutoff of >17 for determining clinical high-risk status. In our sample, 10 participants had ARBS score ≥17, while the remainder had ARBS scores ≤ 13, in the low-risk range. Participants' Barratt impulsivity scale (BIS) scores had a mean of 67.6 ± 16.1 with a range of 44–95. BIS scores range from 30 (least impulsive) to 120 (most impulsive). Normal impulsivity is represented by BIS scores in the 52–71 range, with scores at or below 51 indicating a very controlled individual, and scores at or above 72 indicating high impulsivity (Stanford et al., 2009). Differences in overall BIS scores were driven by all six BIS subscales (see Table 2). BIS and ARBS scores were highly correlated (*r* = 0.78, *p* = 3 × 10^−5^, *t* = 5.42, *df* = 19) as illustrated in Figure 2. BIS scores explained 59.7% of the variance in the ARBS scores.

**Table 2:** **Summary of BIS scores and subscale scores**.

	**Mean ± Std**.	**Min**	**Max**	***R***	***P***
Mean BIS	67.6±16.1	44	95	–	–
BIS 1st order attentional subscale	11.8±4.1	5	19	0.82	4.9 × 10^−6^
BIS 1st order cognitive instability subscale	7.0±1.8	4	10	0.79	1.7 × 10^−5^
BIS 1st order motor subscale	15.2±3.4	11	22	0.78	2.7 × 10^−5^
BIS 1st order perseverance subscale	7.8±2.3	4	11.5	0.78	3.4 × 10^−5^
BIS 1st order self-control subscale	13.8±4.3	7	21	0.83	2.6 × 10^−6^
BIS 1st order cognitive complexity subscale	12.0±3.7	6	19	0.88	1.6 × 10^−7^

*R denotes correlation coefficient comparing subscore against overall BIS score (sum of 1st order subscores). P indicates p-value of statistical test for significant correlation with df = 19*.

### 3.2. Task performance

Participants made commission errors on NoGo trials at a mean rate of 5.8 ± 6.9%, which was low but significantly above zero (*p* < 0.0001, bootstrap test). Error rates did not differ significantly between NoGo trials with aversive vs. neutral distractors (*p* = 0.23, permutation test). NoGo error rates did not vary significantly as a function of participant ARBS risk scores (*p* = 0.36) or Barratt impulsivity scores (*p* = 0.12) on bootstrap regression tests. Fifteen of twenty-one participants made no omission errors on Go trials. Five of twenty-one participants had low omission error rates in the 0.6–3.7% range. One clinical participant fell asleep intermittently toward the end of the study and exhibited an omission error rate of 20.1%. Due to the difficulty of recruiting clinical participants, we did not exclude this participant from the analysis, although we did separate out error trials in the fMRI analysis (see Section 2.6).

Go trial latencies were 772 ± 128 ms with neutral distractors and 808 ± 141 ms with aversive distractors, and this difference was significant (*p* = 0.0003, *t* = 4.7, *df* = 19). Go trial latencies did not show significant relationships with either ARBS risk scores (*p* = 0.42, *t* = 0.82, *df* = 19) or Barratt impulsivity scores (*p* = 0.14, *t* = 1.6, *df* = 19) on linear regression tests. Similarly, Horn et al. (2003) and Asahi et al. (2004) did not find significant relationships between participant impulsivity scores and Go/NoGo task performance.

### 3.3. fMRI main effects results

**Figure 3:**
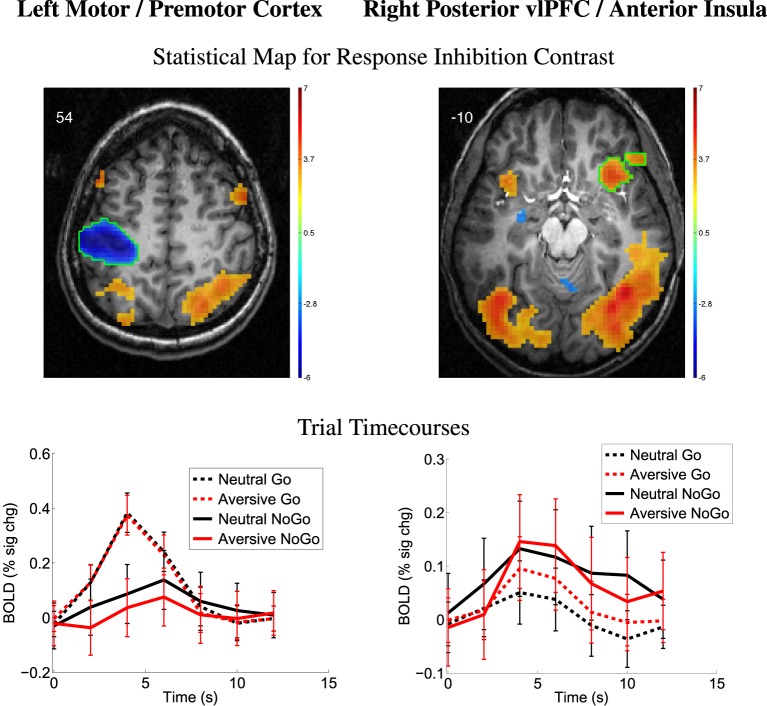
**Top row:** Statistical t-map for response inhibition contrast (NoGo − Go, collapsed across distractor type). Red and blue regions, respectively, exhibited larger contrast magnitudes for NoGo and Go trials. All results *p* < 0.05 (corrected for multiple comparisons). Color bar indicates *t*-value scaling. Slice Z-coordinate in MNI space shown in upper-left. Axial images' left side corresponds to left side of brain. vlPFC: ventrolateral prefrontal cortex. **Second row:** Mean deconvolved timecourses for four trial types for regions outlined in green in first row. Error bars denote mean across all participants' standard error of mean activation time course values.

The response inhibition contrast (NoGo – Go) was significant in many brain regions. Left motor/premotor cortex (Figure 3, left panel), supplementary motor area, posterior insula, and other regions showed greater activation for Go vs. NoGo regions (also see Supplementary Figure 2). The left motor/premotor cortex region shown in Figure 3 exhibited activation significantly above baseline for neutral NoGo trials (*p* < 0.0001, *t* = 5.78, *df* = 20) and non-significantly above baseline for aversive NoGo trials (*p* = 0.11, *t* = 1.69, *df* = 20). Multiple regions exhibited greater activation for NoGo vs. Go trials including right posterior ventrolateral prefrontal cortex (vlPFC)/anterior insula as shown in Figure 3 as well as dorsolateral prefrontal cortex (dlPFC), parietal and occipital regions associated with attention and visual processing, and other regions as shown in Supplementary Figure 2.

**Figure 4:**
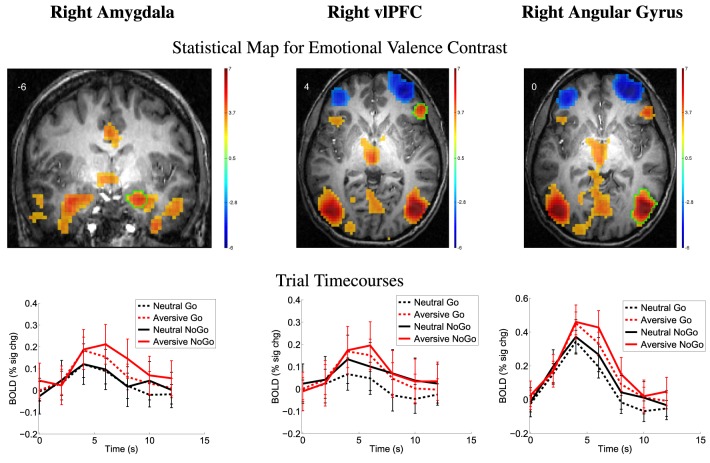
**Top row:** Statistical t-map for emotional valence contrast (aversive − neutral distractors, collapsed across Go/NoGo). Red and blue regions, respectively, exhibited larger contrast magnitudes for aversive and neutral distractor trials. All results *p* < 0.05 (corrected for multiple comparisons). Color bar indicates *t*-value scaling. Slice Y- or Z-coordinate in MNI space shown in upper-left. Images' left side corresponds to left side of brain. vlPFC: ventrolateral prefrontal cortex. **Second row:** Mean deconvolved timecourses for four trial types for regions outlined in green in first row. Error bars denote mean across all participants' standard error of mean activation time course values.

The emotional valence contrast (aversive − neutral distractors) also showed significance in many regions throughout the brain. Multiple regions exhibited positive contrast (aversive > neutral) including the amygdala, vlPFC, and angular gyrus (see Figure 4) as well as supplementary motor area, vlPFC, anterior and posterior cingulate cortex, medial frontal gyrus, orbitofrontal regions, parietal and occipital regions, and anterior temporal regions as shown in Supplementary Figure 3. Several regions showed negative emotional valence contrast (neutral > aversive) including regions in vlPFC, dlPFC, posterior insula, OFC, and other regions (Supplementary Figure 3).

### 3.4. fMRI emotional valence contrast vs. risk and impulsivity scores

**Figure 5:**
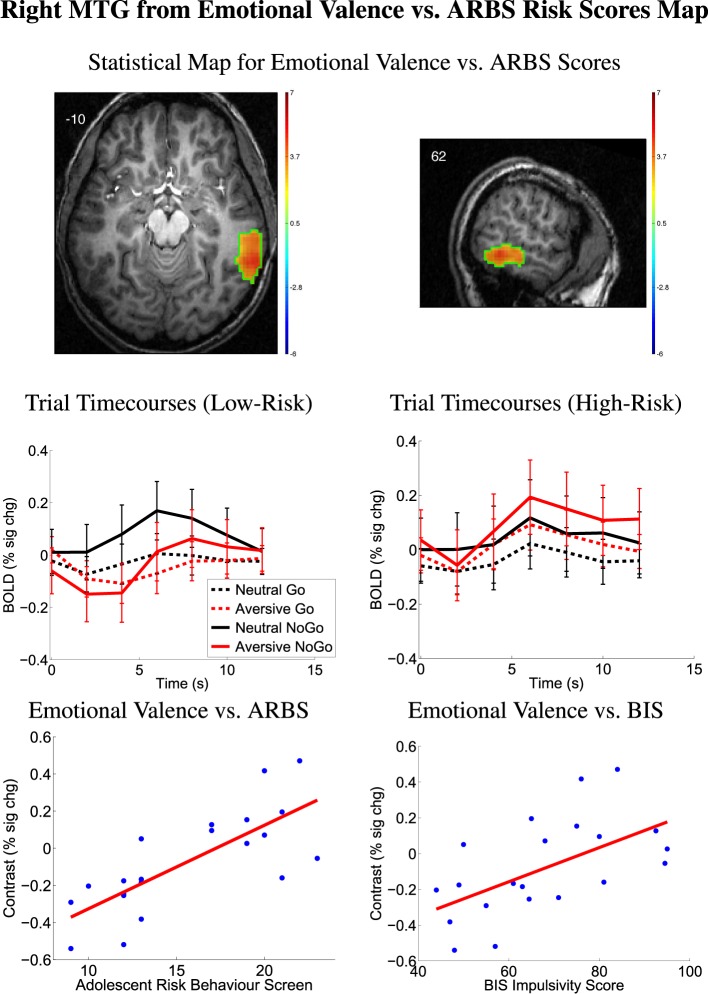
**Top row:** Statistical t-map for regression of ARBS risk scores against fMRI emotional valence contrast (aversive − neutral distractors). Red/yellow regions exhibited larger contrast magnitudes in participants with higher ARBS risk scores. All results *p* < 0.05 (corrected for multiple comparisons). Color bars indicate *t*-value scaling. Slice X- or Z-coordinate in MNI space shown in upper-left. Axial image's left side corresponds to left side of brain. MTG: middle temporal gyrus. **Second row:** Mean deconvolved timecourses for four trial types for region outlined in green in first row. Error bars denote mean across all participants' standard error of mean activation time course values. Timecourses denoted Low-Risk were computed from 11 low-risk participants with ARBS risk scores ≤ 13. Timecourses denoted High-Risk were computed from 10 high-risk participants with ARBS risk scores ≥17. **Bottom row:** Scatter plots show emotional valence contrast magnitude vs. participants' ARBS risk scores (left) and vs. BIS impulsivity scores (right) for right MTG region outlined in green in first row. Red line shows linear regression of contrast magnitude against participant ARBS or BIS scores.

We examined relationships between the emotional valence contrast (aversive − neutral distractor pictures) vs. ARBS risk scores (Map 5). See Section 2.6 for methodological details. There was a positive relationship between emotional valence contrast amplitude and participants' ARBS scores in the temporo-occipital part of right middle temporal gyrus (MTG) (Table 3, Figure 5). Participants with low ARBS scores (≤13) exhibited greater activation for neutral vs. aversive distractor trials resulting in negative values for the first-level (aversive − neutral) contrast. Participants with high ARBS scores (≥17) showed the opposite pattern. The analysis of emotional valence contrast vs. BIS scores (Map 6) did not include a significant cluster surviving multiple comparison correction in right MTG. Follow-up region of interest analysis on the right MTG region from Map 5 did find a significant relationship between emotional valence contrast and BIS scores (Table 3), but caution is recommended in interpreting this result due to potential double-dipping issues (see Kriegeskorte et al., 2009; Vul et al., 2009). This result is explained by the fact that, in Map 6, there is an MTG cluster exhibiting a relationship between emotional valence and BIS scores, but this cluster is too small to survive correction for multiple comparisons. Our results are consistent with, but do not provide conclusive support for, a relationship between emotional valence contrast and BIS scores in right MTG.

**Table 3:** **From emotional valence contrast vs. ARBS score analysis**.

**Region**	***X***	***Y***	***Z***	**Volume**	**Regression vs. ARBS**		**Regression vs. BIS**	
	**(mm)**			**(mm^3^)**	***P***	***T***	***P***	***T***
Right MTG	66.0	–49.0	–11.0	9936	0.001	3.29	0.007	2.76

*Summary data for significant clusters identified in statistical t-maps comparing fMRI emotional valence contrast (aversive − neutral distractors) vs. ARB scores. X, Y, Z: MNI coordinates of region's peak statistical voxel. P- and t-values are median values across all voxels in each region (df = 98). Positive and negative t-values indicate, respectively, greater and lesser emotional valence contrast values for participants with larger ARBS or BIS scores. See Section 2.6 for details of analysis. MTG: Middle Temporal Gyrus*.

### 3.5. Other statistical contrasts

The other statistical contrasts (listed below) did not exhibit any significant regions surviving cluster size threshold correction for multiple comparisons and quality assurance checks (see last paragraph of Section 2.6). The statistical comparisons with no results included:

- **Map 2:** response inhibition contrast vs. ARBS scores,
- **Map 3:** response inhibition contrast vs. BIS scores,
- **Map 6:** emotional valence contrast vs. BIS scores,
- **Map 7:** interaction contrast independent of ARBS or BIS,
- **Map 8:** interaction contrast vs. ARBS scores,
- **Map 9:** interaction contrast vs. BIS scores.

### 3.6. Correlation pattern analysis

**Figure 6:**
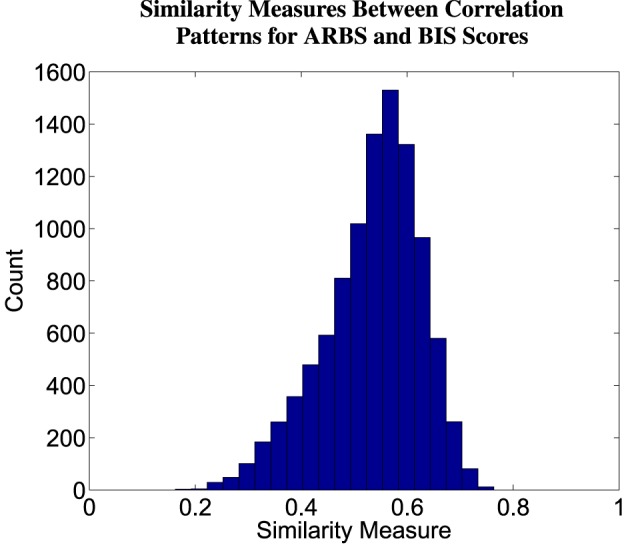
**Histogram of similarity measures between correlation patterns for ARBS and BIS scores**. A correlation pattern captures the relationship between an instrument (ARBS or BIS scores) and fMRI contrast values. See Section 2.7 for details of correlation pattern and similarity measure computation. Values were computed from 10,000 iterations of bootstrap sampling.

For each of 26 prefrontal regions from the Harvard-Oxford atlas, we computed correlations between participant fMRI first-level contrast values and ARBS risk scores as well as BIS impulsivity scores (see Section 2.7). The 26 regions are listed in Table 4. The resulting correlation values comprised a correlation pattern for either ARBS or BIS scores. Table 4 shows correlation patterns computed using all participants. Bootstrap testing with 10,000 iterations was used for statistical testing (see Section 2.7). The mean similarity measure between correlation patterns for ARBS scores and for BIS scores was 0.53±0.09 (std.). A histogram of similarity measures from all iterations is shown in Figure 6. Over all bootstrap iterations, the proportion of similarity measures < 0.3 was 0.01, and the proportion of measures >0.7 was also 0.01. These results indicate that the mean similarity measure was significantly above and below the cutoffs, respectively, defining correlation patterns as substantially different and very similar (see Section 2.7). That is, correlation patterns for ARBS and BIS scores were neither identical (or close to identical) nor completely dissimilar but were instead partially similar while still exhibiting some differences.

**Table 4:** **Results from correlation pattern analysis**.

**#**	**Name**	***X***	***Y***	***Z***	**Volume**	**Corr ARBS**			**Corr BIS**		
						**Inhib**	**Emot**	**Inter**	**Inhib**	**Emot**	**Inter**
1	Left frontal pole	−24	54	8	56079	−0.21	0.08	−0.39	−0.14	−0.12	−0.56
2	Right frontal pole	27	53	9	65097	−0.07	0.27	−0.20	−0.23	0.06	−0.45
3	Left insular cortex	−35	2	1	10530	0.17	0.11	0.16	0.08	−0.13	0.04
4	Right insular cortex	38	4	1	10800	0.04	−0.12	−0.28	0.14	−0.31	−0.32
5	Left superior frontal gyrus	−12	20	57	23571	−0.15	−0.05	−0.24	−0.18	−0.19	−0.42
6	Right superior frontal gyrus	16	19	58	21870	−0.19	0.03	−0.32	−0.18	−0.02	−0.48
7	Left middle frontal gyrus	−37	20	43	23544	−0.24	0.18	−0.37	−0.06	−0.02	−0.47
8	Right middle frontal gyrus	40	20	44	22113	−0.23	0.30	−0.30	−0.20	0.18	−0.33
9	Left inferior frontal gyrus pars triangularis	−49	30	10	5103	0.04	0.13	−0.37	0.11	−0.05	−0.38
10	Right inferior frontal gyrus pars triangularis	53	29	9	4374	−0.21	0.06	−0.18	−0.21	−0.16	−0.37
11	Left inferior frontal gyrus pars opercularis	−50	16	16	6102	0.10	−0.16	−0.23	0.09	−0.35	−0.38
12	Right inferior frontal gyrus pars opercularis	53	17	17	5589	−0.20	−0.30	−0.33	−0.11	−0.34	−0.53
13	Left precentral gyrus	−32	−11	51	35694	−0.10	−0.20	−0.20	−0.16	−0.22	−0.26
14	Right precentral gyrus	35	−10	51	34587	−0.08	−0.22	−0.25	−0.05	−0.33	−0.49
49	Left frontal medial cortex	−3	44	−17	4077	−0.17	0.16	−0.25	−0.42	−0.19	−0.47
50	Right frontal medial cortex	6	43	−18	3834	−0.23	0.07	−0.26	−0.50	−0.21	−0.46
53	Left subcallosal cortex	−3	22	−13	4644	0.03	−0.25	−0.26	0.01	−0.36	−0.32
54	Right subcallosal cortex	6	21	−14	4077	0.14	−0.16	−0.09	−0.01	−0.37	−0.25
55	Left paracingulate gyrus	−5	38	22	11610	−0.37	0.08	−0.22	−0.46	−0.20	−0.50
56	Right paracingulate gyrus	8	38	23	11448	−0.37	0.04	−0.03	−0.51	−0.22	−0.34
57	Left cingulate gyrus anterior division	−3	18	26	10071	−0.20	−0.10	−0.09	−0.28	−0.27	−0.40
58	Right cingulate gyrus anterior division	6	20	25	10908	−0.12	−0.18	−0.10	−0.21	−0.29	−0.37
65	Left frontal orbital cortex	−28	24	−16	13473	−0.05	−0.02	−0.43	−0.04	−0.17	−0.43
66	Right frontal orbital cortex	30	24	−15	11448	0.13	−0.01	−0.31	−0.05	−0.17	−0.40
81	Left frontal operculum cortex	−39	20	5	2889	−0.03	−0.08	−0.04	0.07	−0.25	−0.15
82	Right frontal operculum cortex	42	19	6	2457	−0.29	−0.40	−0.17	−0.04	−0.45	−0.45

*Correlation pattern analysis. 26 regions were used from the Harvard-Oxford atlas. # denotes region numbering from the atlas. X, Y, Z denote region centroid coordinates in mm. Volume is in mm^3^. Correlations between values from each of three first-level fMRI contrasts and either ARBS or BIS scores were computed (denoted Corr ARBS and Corr BIS, respectively). First level contrasts included the response inhibition contrast (denoted Inhib), the emotional valence contrast (Emot), and the interaction contrast (Inter). Also see Section 3.6*.

## 4. Discussion

We investigated fMRI activation in adolescents (age 13–17) performing a Go/NoGo task with emotional distractor images. In the Introduction, we outlined eight specific hypotheses, which are discussed below. Our analyses also included exploratory aspects. In discussing the results from exploratory analyses, we had necessarily to rely on *post-hoc* interpretations, and standard cautions apply, for example related to reverse inference (see Poldrack, 2006). The ultimate test of any result is that it must survive independent replication.

### 4.1. Emotional Go/NoGo task in adolescents

The response inhibition contrast (NoGo − Go) and emotional valence contrast (aversive − neutral distractors) revealed significant differences in many regions throughout the brain (see Section 3.3). These results were mostly similar to those previously reported by Brown et al. (2012) using the same task with young adult participants. Unlike Brown et al. (2012), the current study found regions exhibiting greater activation for neutral vs. aversive distractor images. In dlPFC and vlPFC, Brown et al. (2012) found greater activation for aversive distractors compared to neutral distractors, while the current study found the opposite pattern in dlPFC and parts of vlPFC. This difference may be due to differences in the participant groups. Brown et al. (2012) included young adults age 18–28 with low- to medium-risk behavior tendencies whereas the current study included adolescents age 14–17 with low- as well as high-risk behavior tendencies. The current study's findings are also consistent with previous Go/NoGo studies (Garavan et al., 1999, 2002; Watanabe et al., 2002; Mostofsky et al., 2003; Aron et al., 2004b; Fassbender et al., 2004; Kelly et al., 2004; Rubia et al., 2005; Wager et al., 2005; Aron et al., 2007b; Mitchell, 2011) and with studies of emotional picture processing (Irwin et al., 1996; Bermpohl et al., 2006; Meseguer et al., 2007).

The finding of significant activation above baseline in left motor/premotor cortex for neutral NoGo trials is consistent with a potential role of motor and premotor cortex in response inhibition (see Aron et al., 2007a; Mirabella, 2014). It has been suggested by Mirabella (2014) that initiating a motor response and withholding one may involve interaction among overlapping brain regions based on evaluation of the outcomes of an action (or lack of action). He also proposed that the network of involved brain regions varies based on the decision-making context. For example, the presence of emotional stimuli would recruit emotion processing regions such as the amygdala. This view provides a possible explanation for some regions' involvement in motor control as well as a variety of other tasks. For example, right vlPFC is involved in redirecting selective attention (Corbetta and Shulman, 2002), in motor response inhibition (Aron et al., 2014), and in suppression of memories (Benoit and Anderson, 2012). The overlap between significant regions revealed by our response inhibition and emotional valence contrasts is consistent with this view.

### 4.2. Prefrontal executive control, emotion processing, risk tendency, and impulsivity

Our exploratory statistical comparisons (Maps 1–9, see Section 2.6) did not confirm Hypotheses 1a, 1b, or 1c. We did not find significant prefrontal clusters showing relationships between either ARBS risk scores or BIS impulsivity scores and any of the three first-level contrasts (response inhibition, emotional valence, or interaction contrast). The lack of results may be due to our use of a larger-than-usual cluster size threshold to correct for multiple comparisons in accordance with recent results from Woo et al. (2014) (also see discussion in Section 2.6). Though the large threshold properly corrects for multiple comparisons, it may reduce sensitivity, thereby increasing false negative errors. Our own simulation results (unpublished) support this proposition.

### 4.3. Emotion processing, risk tendency, and impulsivity in MTG

In exploratory analyses, we found that emotional valence contrast values in right MTG were modulated by ARBS risk. High-risk participants exhibited greater right MTG activation for aversive distractor pictures, whereas low-risk participants exhibited greater right MTG activation for neutral distractors. We also found partial but not conclusive evidence for a relationship in right MTG between emotional valence contrast and participant BIS impulsivity scores (see Section 3.4).

Right MTG has been implicated in higher visual processing, response inhibition, and processing emotional stimuli (Schäfer et al., 2005; Sabatinelli et al., 2011; Bhaijiwala et al., 2014). ADHD patients have been shown to exhibit less MTG activity than healthy controls when performing tasks involving inhibition (Bhaijiwala et al., 2014), suggesting that MTG plays a role in inhibition and impulse control disorders. Bhaijiwala et al. (2014) also suggest that right MTG is part of a circuit for task-related functions and inhibition^3^. Associations have also been found between MTG activity, reward processing, and emotional processing. In a meta-analysis, Sabatinelli et al. (2011) located clusters in the MTG that were significantly associated with images of emotional facial expression or emotional scenes, suggesting a role in emotional stimulus processing. Moreover, Schäfer et al. (2005) found increased fMRI activation in MTG for facial expressions and other visual stimuli that elicited fear (as opposed to neutral or disgust-evoking stimuli). They suggest that fear-inducing images may promote a fight-or-flight response involving MTG-based visual processing. Our findings in right MTG are consistent with its suggested role in processing emotional stimuli. Risk- and impulsivity-related modulation of right MTG indicate changes in emotional stimulus processing that may contribute to individual risk tendencies and impulsivity levels.

### 4.4. Impulsivity and high-risk behavior

As indicated by the analysis of correlation patterns (Section 3.6), there were many similarities in the associations that ARBS risk scores and BIS impulsivity scores exhibited with fMRI activation levels related to response inhibition or emotional valence as predicted by Hypothesis 2a. However, the correlation pattern analysis also showed differences between these associations, supporting Hypothesis 2b. We conclude that ARBS risk scores and BIS impulsivity scores show relationships with fMRI activation related to response inhibition and emotional valence that are partially similar while still maintaining differences.

Higher impulsivity scores based on self-report instruments are associated with increased risk behavior tendencies (Levitt, 1991; Moore and Rosenthal, 1993; Luengo et al., 1994; Stanford et al., 1996; Gullo and Dawe, 2008; Romer et al., 2009; Romer, 2010; Dalley et al., 2011; Mishra and Lalumière, 2011; Christiansen et al., 2012; Stautz and Cooper, 2013). However, dissociations between risk behavior and impulsivity have also been reported (Ryan et al., 2013; Brown et al., 2015). The sample of adolescents recruited in the current study exhibited a high correlation (0.78) between ARBS risk scores and BIS impulsivity scores, consistent with a role of impulsivity in contributing to high-risk behavior in the higher risk participants. Accordingly, ARBS risk-related modulation of fMRI activation patterns in the emotional Go/NoGo task were associated with similar BIS impulsivity-related modulation. The relationship between impulsivity and risk behavior is complex and not necessarily consistent (see Romer, 2010; Dalley et al., 2011; Blakemore and Robbins, 2012; Whelan et al., 2012), but our results suggest a relationship between elevated impulsivity and high-risk behavior, both in terms of psychometric testing and modulation of fMRI activation patterns, in groups such as the high-risk adolescent participants included in this study.

### 4.5. Models of risk behavior

Models of high-risk behavior in adolescents proposed by Ernst and colleagues (Ernst et al., 2006; Ernst and Mueller, 2008; Ernst and Fudge, 2009) and by Casey and colleagues (Casey et al., 2008, 2011) posit that limbic responses to emotional stimuli are altered in adolescence, that prefrontal regulatory mechanisms are not fully developed, and that higher rates of risk behavior result from this imbalance. The models differ in details of limbic emotion-related responses. In the Introduction, we outlined Hypotheses 3a and 3b that the models of Ernst et al. and Casey et al. would be consistent with, respectively, reduced and increased emotional valence contrast (aversive − neutral pictures) in limbic regions, particularly in the amygdala, in high-risk participants. Contrary to expectation, we did not observe risk- or impulsivity-related differences in emotional valence contrast in the amygdala, other deep brain nuclei, or other limbic structures associated with emotion processing. As such, our results do not provide differential support for either the Ernst model or the Casey model. We also suggested that both models would be consistent with an association between elevated individual risk behavior tendencies and reduced prefrontal fMRI activation related to response inhibition (Hypothesis 4). Prefrontal regions, such as vlPFC (Figure 3), that exhibited response inhibition-related activation in the form of larger positive BOLD deflections for NoGo vs. Go trials did not exhibit modulation by ARBS or BIS scores. As such, our results do not provide clear support for Hypothesis 4.

### 4.6. Conclusions

We observed partial similarities in how participant risk tendencies and impulsivity levels were associated with changes in fMRI activation patterns related to response inhibition and emotional stimulus processing in prefrontal regions. These changes in activation patterns may reflect changes in processing related to response inhibition and decision-making in emotional contexts that could underlie high-risk behavior tendencies and high impulsivity status.

### Conflict of interest statement

The authors declare that the research was conducted in the absence of any commercial or financial relationships that could be construed as a potential conflict of interest.

## Footnotes

^1^The interaction of response inhibition and emotion processing refers specifically to the interaction contrast (aversive NoGo - aversive Go) - (neutral NoGo - neutral Go). See Section 2.6 for details.

^2^The similarity measure defined in Equation (1) could theoretically assume values in the range [−1, 1]. In our analysis, no similarity measures < 0 were encountered, so we ignored the negative case. Alternatively, one could eliminate the possibility of negative similarity values by truncating negative values to 0.

^3^Although our results included differences related to emotion processing but not response inhibition in right MTG, the results from Bhaijiwala et al. (2014) provide important context in this area.
