# Supplementary material for: fMRI investigation of response inhibition, emotion, impulsivity, and clinical high-risk behavior in adolescents
Source: Front Syst Neurosci. 2015 Sep 29;9:124. doi: 10.3389/fnsys.2015.00124 (PMC4586270; doi:10.3389/fnsys.2015.00124)
Supplement: Supplementary file 1 [file Presentation1.PDF]

Frontiers Systems Neuroscience

Supplementary Material
10 September 2015

Supplementary Material: fMRI investigation of response inhibition, emotion, impulsivity, and clinical high-risk behaviour in adolescents

Matthew R. G. Brown [1,2,*], James R. A. Benoit [1], Michal Juhas [1], Ericson Dametto [1], Tiffanie T. Tse [1], Marnie MacKay [1], Bhaskar Sen [2], Alan M. Carroll [1], Oleksandr Hodlevskyy [1], Peter H. Silverstone [1], Florin Dolcos [1,3], Serdar M. Dursun [1], Andrew J. Greenshaw [1]

[1] *Dept. of Psychiatry, University of Alberta, Edmonton, Alberta, Canada*
[2] *Dept. of Computing Science, University of Alberta, Edmonton, Alberta, Canada*
[3] *Psychology Department, Neuroscience Program, and the Beckman Institute for Advanced Science and Technology, University of Illinois Urbana-Champaign*

Correspondence*:
Matthew R. G. Brown
University of Alberta, Department of Psychiatry, 12-127A Clinical Sciences Building, Edmonton, Alberta, T6G2B3, Canada, mbrown2@ualberta.ca

Reward- and aversion-related processing in the brain: translational evidence for separate and shared circuits

1 IAPS IMAGES

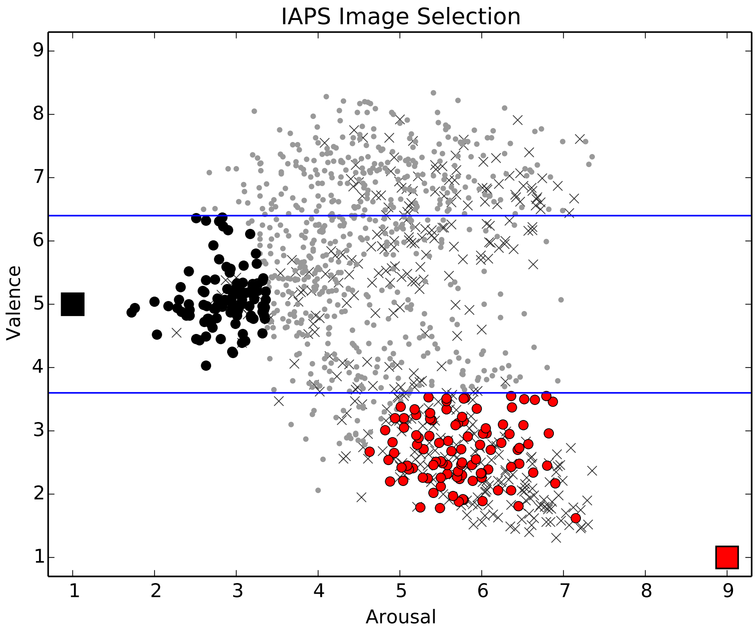

Supplementary Figure 1. Scatterplot of arousal and valence scores for all images in the IAPS picture set. Black and red dots are the images selected as neutral and aversive distractors, respectively. The large black and red squares are the targets for neutral and aversive distractors, respectively (see main text section 2.3). Small gray dots are unused IAPS images. Gray x's are images rejected by the two psychiatrists (see main text section 2.3). Blue horizontal lines mark valence cutoff values of 3.6 and 6.4 for selecting aversive and neutral distractors.

Lists of IAPS images selected for the emotional Go/NoGo task:

Neutral IAPS images: 1333 1450 2002 2036 2038 2102 2104 2190 2200 2210 2320 2357 2381 2393 2396 2397 2411 2440 2480 2491 2493 2495 2499 2570 2580 2620 2745.1 2840 2850 2870 2880 2890 2980 5020 5120 5130 5390 5471 5720 5726 5731 5740 5800 6150 7000 7001 7002 7003 7004 7006 7009 7010 7012 7017 7019 7020 7025 7026 7030 7031 7032 7036 7038 7041 7045 7050 7052 7053 7055 7056 7059 7060 7080 7090 7100 7130 7140 7150 7160 7161 7170 7175 7179 7185 7187 7205 7217 7224 7233 7234 7235 7255 7300 7490 7491 7500 7547 7700 7705 7950 8312 9210 9360 9700

Aversive IAPS images: 1050 1052 1111 1201 1202 1220 1271 1274 1300 1304 1525 2095 2120 2276 2345.1 2375.1 2457 2703 2730 2799 2800 2811 2900 3180 3185 3195 3212 3213 3215 3216 3220 3230 3350 5961 5971 5973 6200 6210 6243 6244 6370 6940 7359 7380 8485 9031 9042 9043 9050 9145 9184 9185 9187 9295 9300 9301 9302 9320 9321 9322 9325 9326 9332 9340 9373 9415 9421 9429 9470 9480 9495 9520 9530 9560 9600 9610 9611 9620 9621 9622 9623 9630 9830 9900 9901 9902 9903 9904 9905 9908 9909 9910 9911 9920 9922 9925 9927 9930 9940 9941

2 SUPPLEMENTARY RESULTS

Response Inhibition Contrast Map

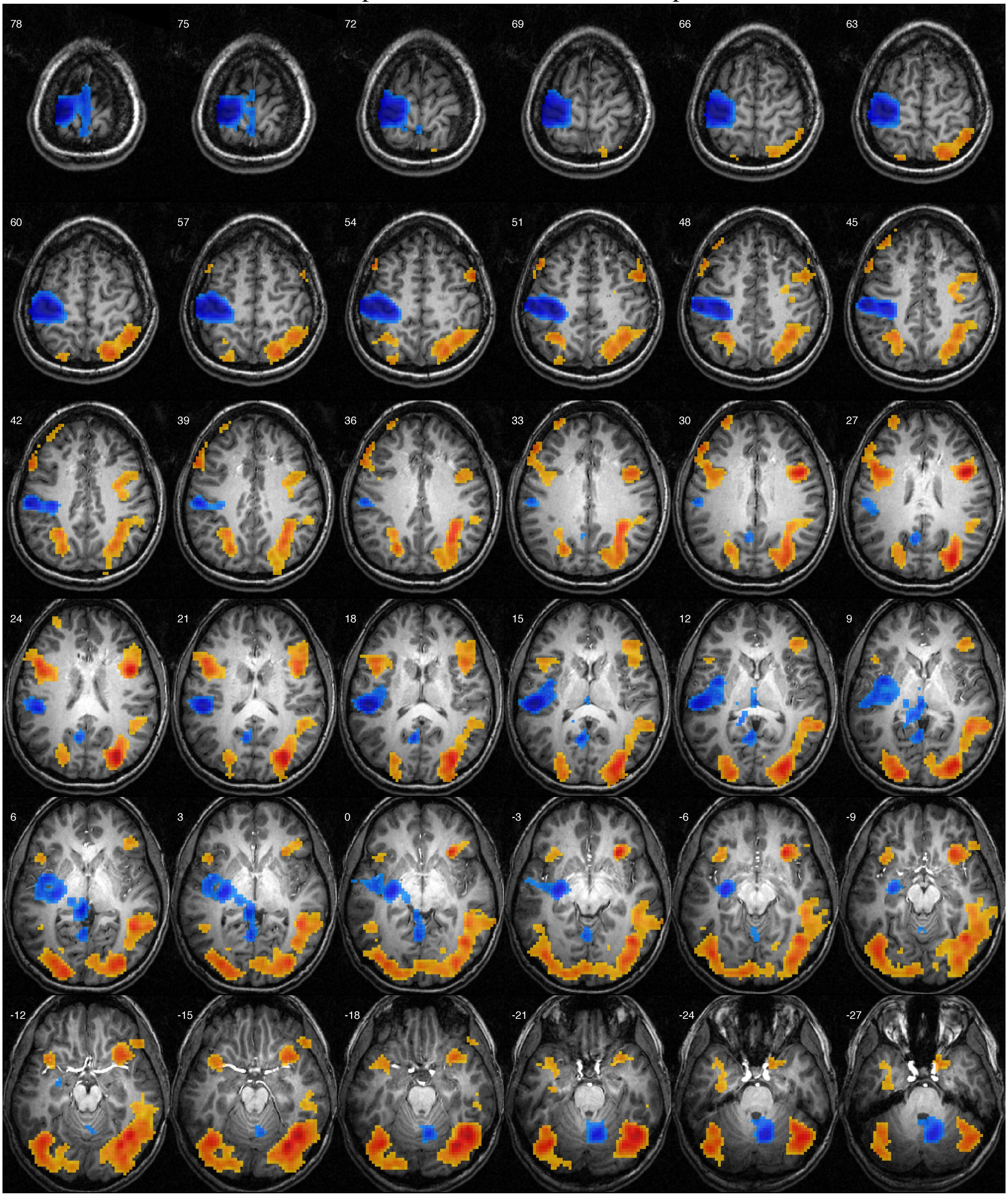

Supplementary Figure 2. Main effects statistical contrast map for response inhibition contrast (NoGo - Go trials), collapsed across aversive and neutral distractors. Neurological convention is used: right side of axial image represents right side of the brain. Yellow/red regions exhibited higher activation for NoGo trials. Blue regions exhibited higher activation for Go trials. Numbers above and left of each slice denote Z coordinates in MNI space in mm. All results $p < 0.05$ corrected for multiple comparisons.

Emotional Valence Contrast Map

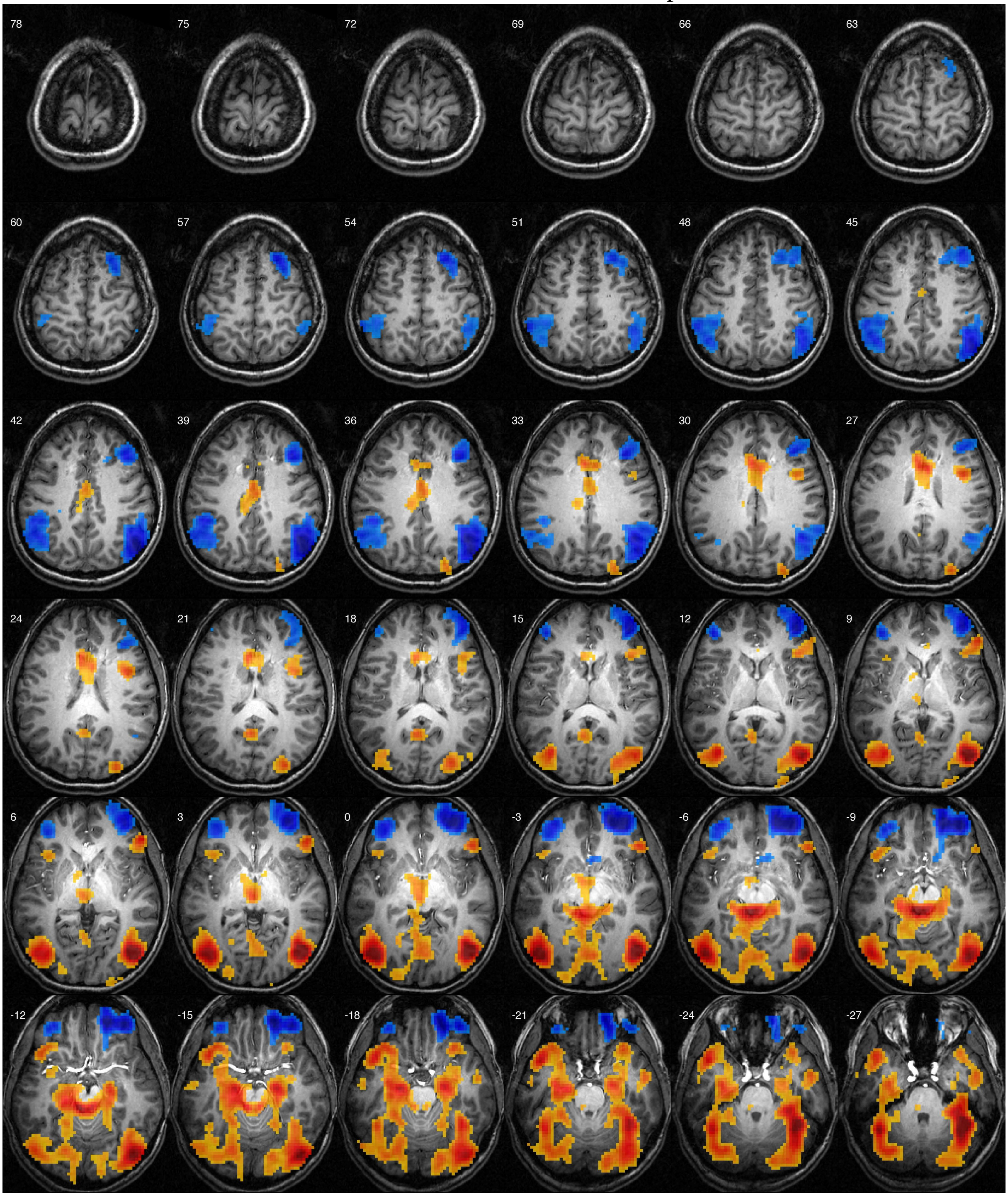

Supplementary Figure 3. Main effects statistical contrast map for emotional valence contrast (aversive - neutral distractor trials), collapsed across Go and NoGo trial types. Neurological convention is used: right side of axial image represents right side of the brain. Yellow/red regions exhibited higher activation for aversive distractor trials. Blue regions exhibited higher activation for neutral distractor trials. Numbers above and left of each slice denote Z coordinates in MNI space in mm. All results $p < 0.05$ corrected for multiple comparisons.
